# Opioid poisoning in Newcastle over the last three decades: From heroin to prescription opioids

**DOI:** 10.1111/1742-6723.14272

**Published:** 2023-07-06

**Authors:** Katherine Z Isoardi, Geoffrey K Isbister

**Affiliations:** ^1^ Clinical Toxicology Research Group The University of Newcastle Newcastle New South Wales Australia; ^2^ Department of Clinical Toxicology Calvary Mater Newcastle Newcastle New South Wales Australia

**Keywords:** codeine, heroin, methadone, opioid poisoning, oxycodone

## Abstract

**Objective:**

Opioid‐related harm has risen in recent decades, but limited research describes the clinical burden of opioid poisoning to Australian EDs. We aimed to investigate hospital presentations with opioid poisoning over three decades.

**Methods:**

This is an observational series of prospectively collected data investigating presentations of opioid poisoning to an ED in Newcastle (1990–2021). Type of opioid, naloxone administration, intubation, intensive care unit (ICU) admission, length of stay and death were extracted from the unit's database.

**Results:**

There were 4492 presentations in 3574 patients (median age 36, 57.7% female), increasing from an average of 93 presentations annually in the first decade to 199 in the third decade. Deliberate self‐poisonings accounted for 3694 presentations (82.2%). Heroin dominated the 1990s, peaking in 1999 before decreasing. Prescription opioids then rose, with codeine (usually in paracetamol combination) predominating until 2018, after which oxycodone presentations exceeded them. Methadone consistently increased from six presentations annually in the first decade to 16 in the last decade. Naloxone was administered in 990 (22.0%) presentations and 266 (5.9%) were intubated, most frequently following methadone and heroin exposures. ICU admissions increased from 5% in 1990 to 16% in 2021. Codeine exposures resulted in less severe effects, whereas methadone had more severe effects overall. The median length of stay was 17 h (interquartile range 9–27 h). There were 28 deaths (0.6%).

**Conclusion:**

Opioid presentations increased in number and severity over three decades as the type of opioid changed. Oxycodone is currently the main opioid of concern. Methadone poisoning was the most severe.

## Graphical abstract

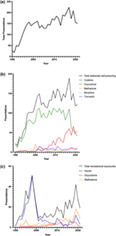

**Key findings**

- This 32‐year observational series saw rising opioid‐related hospital presentations and intensive care unit admissions implying increasing severity and resource burden on the Australian health system.
- Oxycodone is now the primary opioid of concern, overtaking codeine following its up scheduling to a prescription medication.

## Introduction

We are in the midst of a global opioid crisis.^1^ It is estimated that there are 53 million people who misuse opioids annually, representing 1.1% of the world's population aged between 15 and 64 years.^2^ In recent years, opioid‐related mortality has overtaken motor vehicle accidents as the leading cause of traumatic death in many Western countries including the USA, the UK and Australia.^3^

Opioid prescriptions have risen dramatically in Australia over the last few decades, with oxycodone accounting for the majority of this increase, although more recently fentanyl and buprenorphine have contributed to this.^3^ Australia is now the 11th largest pharmaceutical opioid consumer per capita internationally, with this position rising to 3rd and 5th for oxycodone and morphine respectively.^1^ Rising prescriptions have seen a corresponding rise in opioid‐related mortality.^4^ Between 1992 and 2012, opioid dispensing increased from 0.5 to 7.5 million episodes.^3^ This 15‐fold increase was associated with a rise in accidental opioid poisoning deaths from 0.78 to 1.19 deaths/100 000 population.^3^

While there are studies pooling large datasets like prescribing data, hospital separation figures and coronial data to describe the changing epidemiology of opioid‐related harm,^3^, ^4^, ^5^ there is little research describing the clinical burden of opioid poisoning to Australian EDs and hospitals. Furthermore, a recent systematic review identifying studies estimating the prevalence of prescription opioid use in Australia^6^ identified the published literature is limited by incomplete data.

We aimed to describe presentations of opioid poisoning to an ED over a 32‐year period and identify temporal trends, particularly related to specific opioids and poisoning severity.

## Methods

### Study design and setting

This is an observational series of prospectively collected data investigating presentations of opioid poisoning to an ED over a 32‐year period. The Hunter Area Toxicology Service, based at the Calvary Mater Newcastle hospital, was established in 1987 and provides a comprehensive 24 h a day toxicology treatment service for those presenting to the ED with poisoning. It has direct clinical responsibility for all poisoned patients presenting to the Calvary Mater Newcastle and provides a consultative service for the greater Newcastle area, servicing a population of about 500 000. Data on every patient presentation to the unit are entered prospectively into a relational database capturing information on demographics, exposure details, clinical findings, investigations, management and complications. Admission data are collected on either a preformatted admission sheet or tablet‐based application,^7^, ^8^ with all cases reviewed weekly by a clinical toxicologist.

### Selection of participants

Adult patient (>15 years) presenting with an opioid exposure between January 1990 and December 2021 were included in the study. Opioid exposure was based on patient history and examination as part of the toxicological assessment.

### Data collection

We extracted data for all opioid exposures from the toxicology database. This included patient characteristics (age, sex), exposure details (presentation date, opioid, dose, co‐ingestants, intent), management (naloxone, intubation), complications (seizure, death), intensive care unit (ICU) admission and length of stay.

### Analysis

Descriptive statistics were used with continuous variables reported as medians, interquartile ranges (IQRs) and ranges. To compare the doses ingested for differing opioids, an oral morphine equivalent dose (OME) was calculated using the table synthesised by Nielsen *et al*.^9^ with multiplication factors of the following: parenteral buprenorphine × 75, sublingual buprenorphine × 38.8, codeine × 0.1, oral dextropropoxyphene × 0.1, parenteral fentanyl × 0.2, transdermal fentanyl × 2.7, oral hydromorphone × 5, parenteral morphine × 3, oral oxycodone × 1.5, parenteral pethidine × 0.4, oral tapentadol × 0.4 and oral tramadol × 0.4. Dose equivalents for heroin and methadone were not performed. All analyses were performed in GraphPad Prism 9.0.1 for Mac OS (GraphPad Software, San Diego, CA, USA).

### Ethical considerations

The local human research ethics committee has granted an exemption regarding use of the database and patient information for research.

## Results

There were 4492 presentations in 3574 patients over the period. There were 918 representations (median of 1 representation, range 1–28 representations). The median age was 36 years (IQR 26–46 years; range 16–97 years) with a small female preponderance of 2067 (57.8%) (Table 1).

**TABLE 1:** Baseline characteristics of 4492 presentations of opioid poisoning to the Hunter Area Toxicology Service from 1990 to 2021

Number of presentations	4492
Number of patients	3574
Number of representations	918
Median number of representations (range)	1 (1–28 representations)
Median age in years (IQR) [range]	36 (26–46) [16–97]
Sex, *n* (%)	
Female	2067 (57.8)
Male	1502 (42.0)
Transgender	5 (<0.1)
Intent of poisoning, *n* (%)	
Deliberate self‐poisoning	3694 (82.2)
Recreational	617 (13.7)
Unintentional	142 (3.2)
Iatrogenic	22 (0.5)
Adverse reaction	2 (<0.1)
Unknown	15 (0.3)
Co‐ingestions†, *n* (%)	4023 (89.6)
Paracetamol	2439 (54.3)
Benzodiazepines	1335 (29.7)
Ethanol	1174 (26.1)
Doxylamine	534 (11.9)
Ibuprofen	439 (9.8)

† In 1884 of 4023 presentations, there were multiple agents co‐ingested.

**Figure 1:**
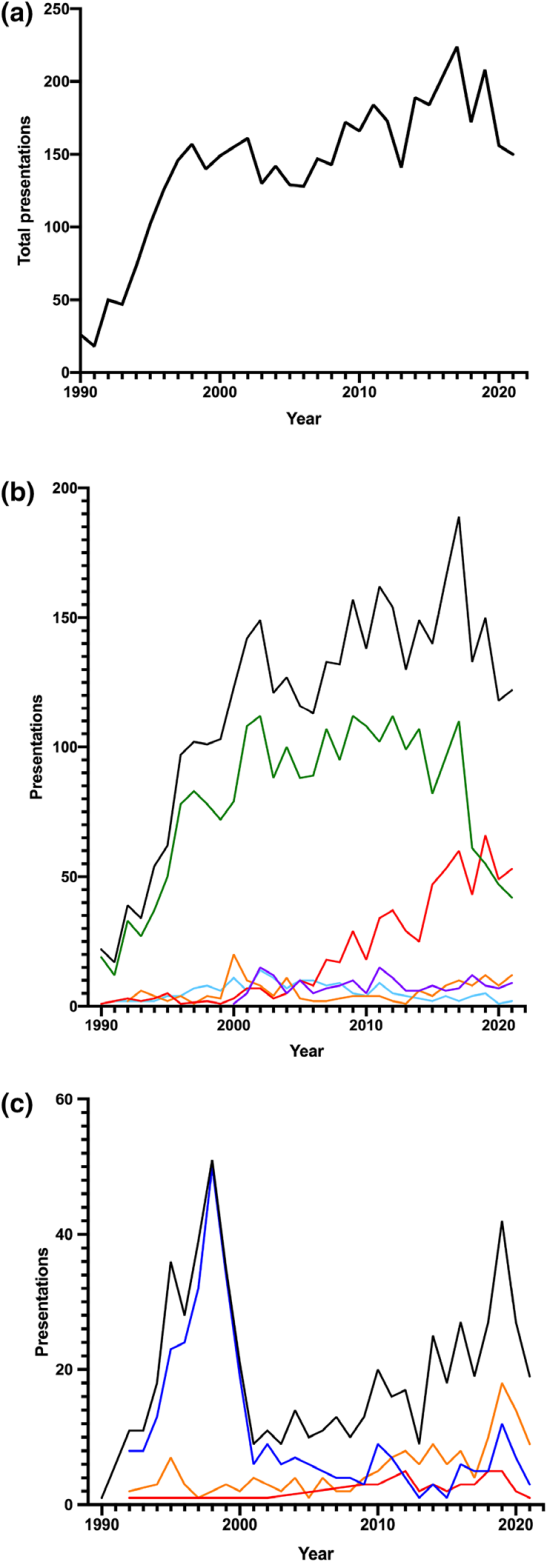
Opioid presentations to Hunter Area Toxicology Service from 1990 to 2022, (a) total presentations to the unit over time, (b) deliberate self‐poisoning presentations over time separated by specific opioid (codeine, oxycodone, heroin, methadone and tramadol), (c) recreational exposures over time separated by specific opioid (heroin, oxycodone and methadone). (b) (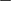), Total delibetate self‐poisoning; (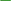), codeine; (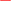), oxycodone; (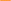), methadone; (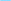), morphine; (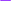), tramadol. (c) (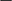), Total recreational exposures; (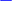), heroin; (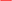), oxycodone; (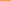), methadone.

Opioid‐related presentations rose consistently from an average of 93 presentations each year in the first decade to 199 in the third decade. Presentations were largely following deliberate self‐poisonings, except in the late 1990s when recreational heroin predominated (Fig. 1). Co‐ingestants were taken in 4023 (89.6%) presentations, most commonly paracetamol combination analgesics with codeine, then other sedatives such as benzodiazepines and ethanol being the next most common co‐ingestants (Table 1).

The commonest opioid exposure was codeine taken in 2618 (58.3%) presentations, and in 2298 of 2618 cases (87.8%) paracetamol was co‐ingested as a paracetamol‐codeine combination. Oxycodone (724 [16.1%]), heroin (496 [11.0%]) and methadone (347 [7.7%]), were the next three most common (Table 2). Codeine presentations steadily increased from 21 in 1990 to a peak of 121 presentations annually in 2017 (Fig. 1). Codeine presentations then rapidly decline from 2018. Heroin presentations were common in the first decade, with a spike late in the 1990s (peak of 63 presentations in 1998), which then dropped in 2000. Oxycodone presentations became more common from 2009 and overtook codeine as the most common opioid exposure in 2018. Methadone presentations, particularly recreational exposures, consistently increased from six presentations each year in the first decade to 16 in last decade.

**TABLE 2:** Opioid ingested and features of severe toxicity in 4492 opioid overdose presentations over a 32‐year period (1990–2021)

	*n* (%)
Opioid ingested	
Codeine	2618 (58.3)
Oxycodone	724 (16.1)
Heroin	496 (11.0)
Methadone	347 (7.7)
Morphine	206 (4.6)
Tramadol	195 (4.3)
Dextropropoxyphene	48 (1.1)
Fentanyl	43 (1.0)
Tapentadol	39 (0.9)
Buprenorphine	37 (0.8)
Hydromorphone	15 (0.3)
Pethidine	12 (0.3)
Dextromethorphan	4 (<0.1)
Dextromoramide	4 (<0.1)
Dihydrocodeine	2 (<0.1)
Hydrocodone	1 (<0.1)
Unknown opioid	39 (0.9)
Multiple opioids ingested	313 (7.0)
Severe toxicity	
Naloxone administration	990 (22.0)
Intubation	266 (5.9)
Seizure	62 (1.4)
ICU admission	354 (7.9)
Death	28 (0.6)
Median length of stay (IQR), h	17 (9–27)

IQR, interquartile range.

Dose information was available for 3143 individual opioid exposures. The total median OME was 47 mg, with individual opioids having a range of median doses from the highest median of 820 mg in hydromorphone exposures and the lowest of 31 mg in codeine exposures. The total median OME over time did not change significantly over the period (Fig. S1).

Naloxone was administered in 990 (22.0%) presentations. It was more often given following exposures to fentanyl (26/43 [60.4%]), methadone (207/347 [59.6%]) and heroin (293/496 [59.1%]). Endotracheal intubation was undertaken in 266 (5.9%) presentations. Seizures were uncommon, occurring in 62 (1.4%) presentations, but were more common in tramadol exposures, in which they occurred in 18 of 195 (9.2%) presentations.

**Figure 2:**
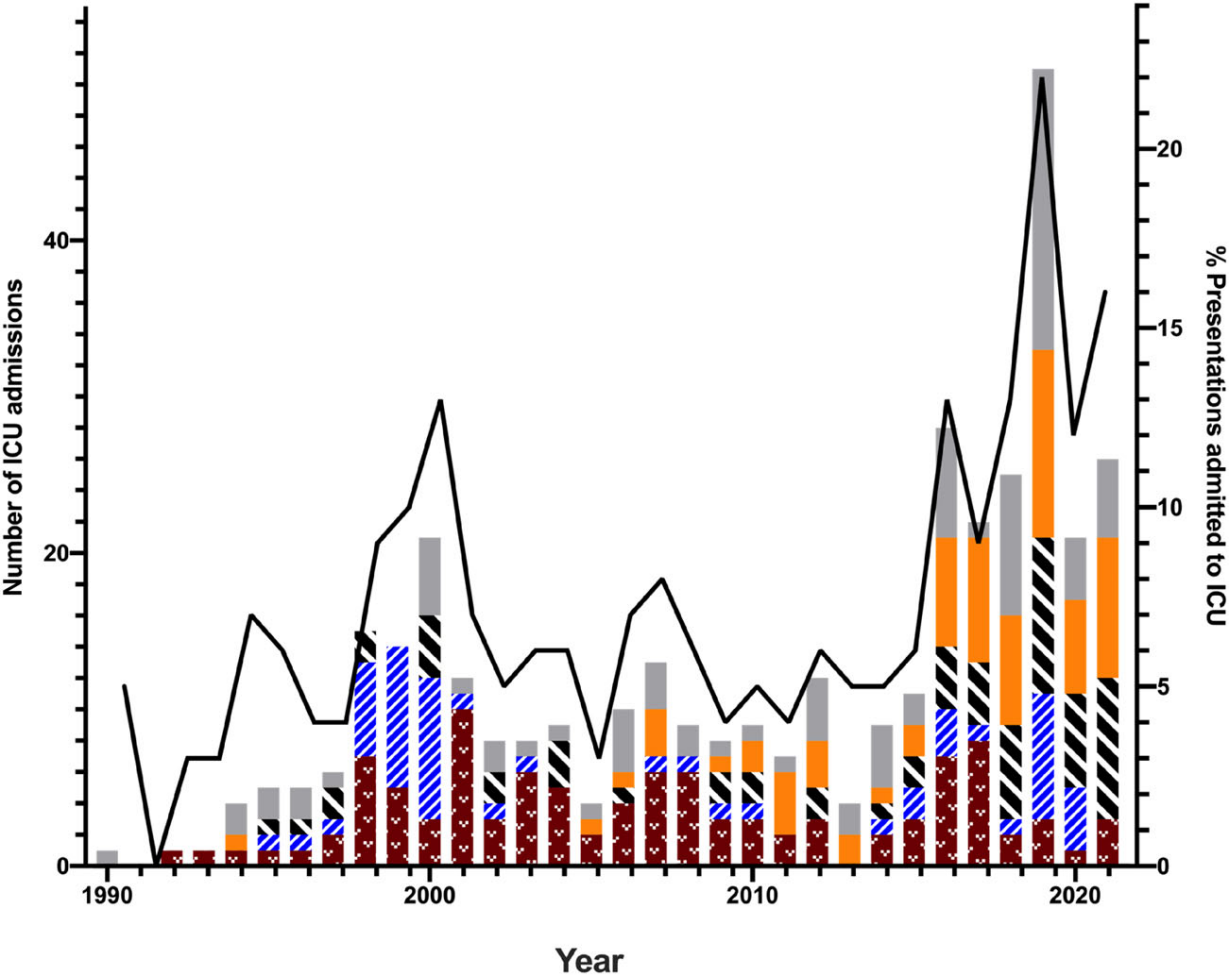
Intensive care unit (ICU) admission following opioid presentations to Hunter Area Toxicology Service from 1990 to 2022. Columns represent number of ICU admissions for specific opioids (left Y axis). Black line represents proportion of total opioid presentations admitted to the ICU (right Y axis). (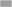), Other opioid; (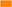), oxycodone; (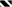), methadone; (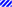), heroin; (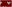), codeine.

Admission location information was available in 4393 presentations. There were 354 (7.9%) presentations managed in the ICU. Most patients (3991 [88.8%]) were managed entirely in the ED (including the short stay unit) with only 48 patients being admitted to a medical ward. ICU admissions following opioid poisoning rose over the period (Fig. 2) from 5% in 1990 to 16% in 2021. There appeared to be two peaks in ICU admissions over the study period, the first in 2000 at 13%, driven largely by heroin poisoning and a more recent larger peak of 22% in 2019 where oxycodone and methadone poisoning predominate. The median length of stay was 17 h (IQR 9–26 h). There were 28 deaths in the series (Table 3).

**TABLE 3:** Features of the six most common individual opioids taken in overdose over a 32‐year period (1990–2021)

Opioid	Codeine	Oxycodone	Heroin	Methadone	Morphine	Tramadol
Presentations	2618	724	496	347	206	195
Deliberate self‐poisoning, *n* (%)	2488 (95)	639 (88)	169 (34)	172 (50)	165 (80)	178 (91)
Co‐ingestions, *n* (%)	2608 (99)	644 (89)	322 (65)	255 (73)	177 (86)	168 (86)
Naloxone, *n* (%)	171 (7)	205 (28)	293 (59)	207 (60)	106 (51)	22 (11)
Intubation, *n* (%)	106 (4)	44 (6)	43 (9)	31 (9)	28 (14)	12 (6)
Seizure, *n* (%)	19 (<1)	4 (<1)	16 (3)	7 (2)	2 (1)	18 (9)
Death, *n* (%)	6 (<1)	0	9 (2)	2 (1)	8 (4)	0
ICU admission, *n* (%)	108 (4)	70 (10)	55 (11)	64 (18)	39 (19)	12 (6)
Median LOS, h	16	17	12	19	24	16

## Discussion

Opioid poisoning presentations increased over the three decades and were largely a result of deliberate self‐poisonings of pharmaceuticals. However, the trends over time for different opioids were not simple, being influenced by the availability of different prescription and illicit opioids. Codeine was the most common overall, principally due to its availability in combination with paracetamol. Codeine exposure frequency is likely to reflect the importance of paracetamol poisoning, and to a lesser extent ibuprofen. In contrast, oxycodone is not a combination agent and reflects increasing use and misuse of prescription opioids – the opioid crisis.

The severity of opioid poisoning presentations appears to have increased in recent years with more patients admitted to the ICU for management, despite no or little increase in the median average dose ingested. This appears to reflect the switch from the weak opioid codeine to the more potent prescription opioid oxycodone and increasing recreational methadone poisoning (Figs 1b,2). ICU admissions, naloxone use and deaths were lowest with codeine, and this is despite a likely overestimation of ICU admissions and deaths, because much of this morbidity is a result of the co‐ingested paracetamol toxicity. A previous study demonstrated the minimal toxicity of codeine in paracetamol combination overdoses.^10^ In contrast, the severity with oxycodone, heroin and methadone is directly related to their pharmacokinetics and opioid agonist potency.

The temporal trends in our series of poisoning by individual opioids are similar to that previously reported in state‐wide and national datasets. The prominence of heroin in the first decade of this series reflects the strong heroin market of the 1990s, with the availability of low‐cost, high‐purity heroin in New South Wales driving heroin being the main substance of concern in people using intravenous drugs.^11^ A sudden decrease in presentations related to heroin follows a well‐documented heroin shortage in 2001.^11^ This decrease was sustained, with oxycodone presentations overtaking heroin presentations in 2006 (Fig. 1).

The rise of oxycodone prescriptions and oxycodone‐related harm in Australia has been previously described. One cross‐sectional study^12^ analysing prescription and hospital separation data found that over the period of 2002 to 2008 oxycodone prescriptions increased by 152%, with a near doubling of hospital separations for poisoning with prescription opioids. Similarly, a recent Australian series of Victorian ED administrative data from 2009 to 2019 found codeine and oxycodone to be the commonest opioids taken in overdose accounting for 37% and 29% of opioid overdose presentations respectively.^13^ Two Australian studies, one in New South Wales and one in Victoria, found a significantly increased in deaths from oxycodone poisoning from 1999 to 2019,^14^, ^15^ associated with an increase in supply in the Victorian study.^16^

The Australian experience with opioids has been likened to that of the USA, albeit 10 years earlier.^17^, ^18^ An important difference is that Australia has not yet experienced an increase in fentanyl and fentanyl derivative overdoses, which account for the bulk of morbidity and mortality in the USA. Fentanyl‐related presentations were uncommon in our series accounting for only 1% of presentations (Table 2).

The rising severity of opioid poisoning in patients presenting to EDs is concerning, with a greater proportion of patients being admitted to the ICU in recent years. This most likely reflects the predominance of relatively more potent opioids, importantly oxycodone and methadone, compared to the low‐potency opioid codeine. Our series demonstrated a clear decrease in codeine poisoning presentations when codeine was up scheduled by the Therapeutic Goods Association in February 2018 to prescription only. This experience was mirrored in other toxicology units across Australia^19^ and supports a role for tighter restrictions on opioid scheduling in an effort to curb misuse. It is possible other harm mitigation measures including the introduction of real‐time prescription monitoring for opioid medications, which has been introduced across all states of Australia over the last 2 years, may also have a positive impact.^20^

A number of other prescription opioids were less commonly taken in overdose. Tramadol was the fifth most common opioid taken in overdose and contributed to the rise in opioid deliberate self‐poisoning slightly earlier than oxycodone in 2000. Tramadol overdose had the highest incidence of seizures and is also associated with serotonin toxicity.^21^ Tapentadol is another important opioid, and recent data suggests it may soon become one of the most common opioids ingested in overdose.^22^, ^23^

This series has some important limitations. Although we used a prospective cohort, which is reviewed weekly by a clinical toxicologist, the present study retrospectively reviewed the presentations. Furthermore, data on ingested drugs were based on patient history and did not have any confirmatory analytical testing, although accuracy in patient history following overdose is supported by pharmacokinetic studies of drug overdose.^24^, ^25^ Another limitation is that we used naloxone as a marker of severity as its indication is to reverse respiratory depression or coma following opioid overdose. Naloxone use is clinician dependent, so it is possible some may have given naloxone to patients without severe toxicity; however, we feel this effect would be limited given the oversight of the clinical toxicology service in the ED. The lethality of opioid poisoning is likely to be underestimated in this series,^4^ given the present study only included patients who reached hospital. Finally, the present study reports the experience of a single urban centre and may not be representative of other units across Australia, and importantly different inner city and regional populations.

## Conclusion

Opioid poisoning presentations have risen over the last three decades. Deliberate self‐poisonings of pharmaceuticals account for most opioid presentations presenting to hospital. Oxycodone is currently the main opioid of concern, overtaking codeine in recent years. Concerningly, the severity of poisoning appears to be rising with the proportion of patients admitted to ICU increasing threefold. Measures to limit access to prescriptions opioids may have an important role to mitigate harm related to prescription opioids.

## Supporting information


**Figure S1.** Median oral morphine equivalent dose (OME) of opioid presentations to the Hunter Area Toxicology Service from 1990 to 2022.

## Data Availability

Data available on request from the authors.
