# Supplementary material for: Opioid poisoning in Newcastle over the last three decades: From heroin to prescription opioids
Source: Emerg Med Australas. 2023 Jul 6;35(6):946–52. doi: 10.1111/1742-6723.14272 (PMC10947284; doi:10.1111/1742-6723.14272)

Supplementary Figure:

Median oral morphine equivalent dose (OME) of opioid presentations to the Hunter Area Toxicology Service from 1990 to 2022.


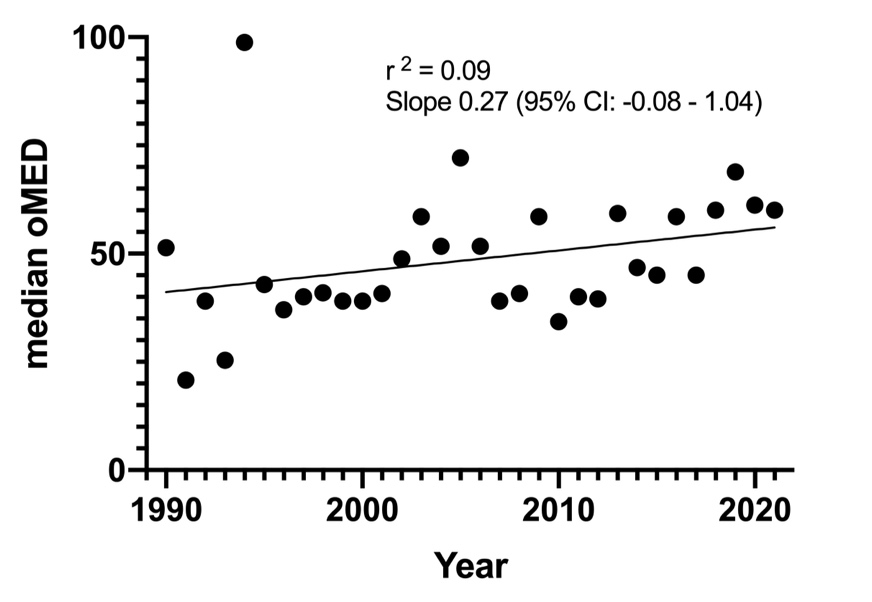

Supplement: Supplementary file 1 — Figure S1. Median oral morphine equivalent dose (OME) of opioid presentations to the Hunter Area Toxicology Service from 1990 to 2022. [file EMM-35-946-s001.docx]
